# Comparison of Blue Light-Filtering IOLs and UV Light-Filtering IOLs for Cataract Surgery: A Meta-Analysis

**DOI:** 10.1371/journal.pone.0033013

**Published:** 2012-03-07

**Authors:** Xiao-feng Zhu, Hai-dong Zou, Yong-fu Yu, Qian Sun, Nai-qing Zhao

**Affiliations:** 1 Department of Ophthalmology, Shanghai First People's Hospital Affiliated Shanghai Jiao Tong University, Shanghai, China; 2 Department of Biostatistics, School of Public Health, Fudan University, Shanghai, China; University of York, United Kingdom

## Abstract

**Background:**

A number of published randomized controlled trials have been conducted to evaluate visual performance of blue light-filtering intraocular lenses (IOL) and UV light-filtering intraocular lenses (IOL) after cataract phacoemulsification surgery. However, results have not always been consistent. Therefore, we carried out a meta-analysis to compare the effectiveness of blue light-filtering IOLs versus UV light-filtering IOLs in cataract surgery.

**Methods and Findings:**

Comprehensive searches of PubMed, Embase, Cochrane Library and the Chinese BioMedical literature databases were performed using web-based search engines. Fifteen trials (1690 eyes) were included for systematic review, and 11 of 15 studies were included in this meta-analysis. The results showed that there were no significant differences in postoperative mean best corrected visual acuity, contrast sensitivity, overall color vision, or in the blue light spectrum under photopic light conditions between blue light-filtering IOLs and UV light-filtering IOLs [WMD = −0.01, 95%CI (−0.03, 0.01), P = 0.46; WMD = 0.07, 95%CI (−0.04, 0.19), P = 0.20; SMD = 0.14, 95%CI (−0.33, 0.60), P = 0.566; SMD = 0.20, 95%CI (−0.04, 0.43), P = 0.099]. However, color vision with blue light-filtering IOLs was significantly reduced in the blue light spectrum under mesopic light conditions [SMD = 0.74, 95%CI (0.29, 1.18), P = 0.001].

**Conclusion:**

This meta-analysis demonstrates that postoperative visual performance with blue light-filtering IOLs is approximately equal to that of UV light-filtering IOLs after cataract surgery, but color vision with blue light-filtering IOLs demonstrated some compromise in the blue light spectrum under mesopic light conditions.

## Introduction

Globally, cataract is one of the most serious blinding diseases [1]. Modern cataract surgery is routinely combined with the implantation of an intraocular lens (IOL). UV light-filtering lenses have been the dominant IOLs used in modern cataract surgery since the mid-1980 s because of the growing evidence that ultraviolet light caused photic retinopathy and cystoid macular edema [2]. Recently there has been support for increasing the absorption spectrum of IOLs. The rationale is that UV light-filtering IOLs do not protect the retina from phototoxic damage by high-energy, short-wavelength blue light (approximately 400–480 nm) which is thought to contribute to the pathogenesis of age-related macular degeneration (AMD) [3], [4]. The healthy human crystalline lens gradually becomes yellow as part of the normal ageing process. This yellowing reduces the transmission of blue light, thereby blocking an amount of blue light from reaching the retina [5]. After cataract extraction, the possibility of retinal exposure to blue light may accelerate AMD [6]. To address this potential damage, several blue light-filtering IOLs have been introduced in recent years. Their yellow tint more closely replicates the spectral transmission properties of the aged human crystalline lens than do the UV light-filtering IOLs [7].

Despite the benefits of blue light filtering, concerns were raised that this could negatively affect visual performance after cataract surgery [8]. Specifically, controversy remains as to ultimate best corrected visual acuity (BCVA), contrast sensitivity, color vision and glare. In recent years, several studies were performed to test visual function with blue light-filtering IOLs. Some described a better [9]–[11] and others, a worse [8], [12], [13], outcomes. However, we find that the majority of the literature did not describe any statistically significant differences in comparisons with UV light-filtering IOLs [14]–[17], or, if present, these differences were most likely small.

In this paper, we conduct a systematic review and meta-analysis of published randomized controlled trials to assess the visual performance of blue light-filtering IOLs and UV light-filtering IOLs after cataract surgery.

## Materials and Methods

### 1. Search strategy

Two independent investigators (Zhu and Yu) searched publications from 2000 to June 30th, 2011 in PubMed, Embase, Cochrane Library, and the Chinese BioMedical Literature (CBM) databases by using the combination of MeSH terms “cataract extraction” or “phacoemulsification” or “lens” or “intraocular” or “implantation” or “blue light filtering” or “blue blocking” or “AcrySof Natural” or “SN60AT” or “yellow intraocular lens” or “YA60BB”. In addition, the reference lists of potentially relevant manuscripts were scanned backwards to obtain extra eligible studies. No language restrictions were applied.

### 2. Inclusion criteria

**2.1. Type of study:** For inclusion, studies had to be randomized controlled trials (RCTs) comparing postoperative visual performance of blue light-filtering IOLs and UV light-filtering IOLs. Simulation experiments with blue light-filtering IOLs and UV light-filtering IOLs and clinical trials containing aspherical or multifocal IOLs were excluded.

**2.2. Object of study:** Patients with age-related cataract had phacoemulsification and IOL implantation. Otherwise, patients with ocular disease, such as glaucoma or age-related macular degeneration, preexisting systemic disease such as diabetes, or history of intraocular surgery that could affect the postoperative visual outcome were excluded.

**2.3. Interventions:** In addition to the different types of intraocular lens (blue light-filtering IOL or UV light-filtering IOL) implanted in the two groups, the interventions were the same.

**2.4. Outcome measures:** Main outcome measures included postoperative best corrected visual acuity, contrast sensitivity and color vision. Secondary outcome measures included postoperative visual quality assessment and adverse visual events.

### 3. Data extraction

Two independent investigators (Zhu and Yu) were involved in data extraction. The third investigator (Zou) examined the results, and a consensus was reached. The outcome in patients at the end of follow-up after phacoemulsification and IOL implantation was reviewed. We extracted the following data from the eligible studies: (1) general characteristics (title, first author, journal and year of publication); (2) methodology (type of study, country of origin, sequence generation, allocation concealment, masking or blinding, incomplete outcome data, selective reporting and other sources of bias); (3) subjects (recruitment site, enrollment periods, inclusion criteria, exclusion criteria, general patient characteristics); (4) Interventions and control groups (model of IOLs); (5) outcomes (measurement, follow-up time and loss of follow-up); (6) analysis (statistical methods); (7) results (quantitative results, qualitative results, postoperative visual quality assessment and adverse visual events). If original data were unavailable in articles, a request for original data was sent to the corresponding author.

### 4. Assessment of methodology quality

Two independent investigators (Zhu and Yu) evaluated the quality of each study using the Jadad scale [18]. The third investigator (Zou) examined the results, and a consensus was reached. The Jadad score is obtained from a possible 5-point scale, high scores indicating high quality, by yes/no answers to two questions for randomization and masking, and one question evaluating the reporting of patient withdrawals and dropouts. One point is given for each of the following: if the study is described as randomized, if the study is described as double-blind and if there is a description of withdrawals or dropouts. Two additional points are given if the method of randomization and the method of double blinding are appropriately described.

### 5. Statistical analysis

The meta-analysis was performed with the Stata version 11.0 (Stata Corp). For continuous data, the weighted mean difference (WMD) and 95%CI were recommended when all trials used the same scale to report their outcomes, while standardized mean difference (SMD) and 95%CI were more appropriate when trials used different scales to report their outcomes, or the means of their outcomes differed greatly. For dichotomous data, rate ratio or relative risk (RR) was strongly recommended for effect statistics for meta-analysis of randomized trials.

Statistical heterogeneity was tested by Q-test (x^2^) [19] for each outcome, with a significance set at a P value<0.10. Insignificance indicates that the results of the different trials were similar (P≥0.1, I^2^≤50%). We evaluated the pooled summary effect by using a fixed-effect model to reduce the effects of heterogeneity between trials. Otherwise, data were combined using the random-effect model (P<0.1, I^2^>50%). If I^2^>75%, subgroup analysis was used to analysis the sources of heterogeneity. Sensitivity analysis calculated within subgroups of studies decided a priori were performed to assess the robustness of the main conclusions and explain heterogeneity. To determine whether the results of the meta-analysis were unduly influenced by any outcome measures in any one study, we recomputed the meta-analysis statistic after deleting each outcome measure one at a time.

Individual and pooled results were illustrated by point estimates and 95% confidence intervals (CIs). Results where the 95% CI did not include zero (for mean difference) or one (for odds ratio) were considered statistically significant. Where data could not be combined, we conducted a descriptive analysis.

## Results

### 1. Literature search

**Figure 1:**
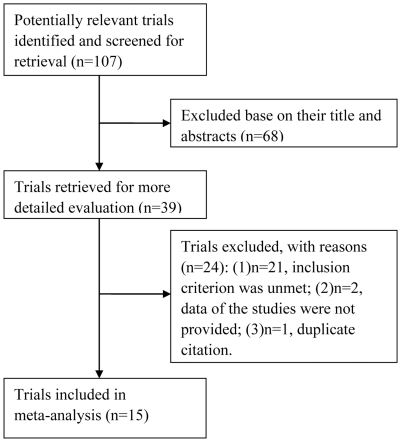
Process of study selection of RCTs.

A total of 107 abstracts from the multiple databases were retrieved, 68 of which were based on their titles and abstracts. Only 15 randomized controlled trials (RCTs) [14], [17], [20]–[32] recruiting 1690 eyes were included in our analysis. The trials selection process is shown in Figure 1.

### 2. Characteristics of eligible studies

Characteristics of RCTs included in the current meta-analysis are presented in Table 1 and Table 2. More than half of the studies [20], [21], [17], [23], [24], [26], [28], [29], [31] (60%, 9/15) were performed in Europe followed by 4 studies [14], [22], [30], [32] in Asia; one study [25] from Australia and one study [27] from Brazil. The mean age of patients in most of the studies ranged from 50 to 88 years. The mean percentage of female patients ranged from 48.51% to 75%, as shown in Table 1. The mean follow-up time ranged from 2 months to 2 years (Table 2).

**Table 1:** Characteristics of RCTS (n = 15) included in the meta-analysis.

First author (date)	Site	No. patients	No. eyes	Age (years)	Sex(%) male: female	Intervention
				(test group/control group)		
Hayashi 2006 [14]	Japan	80	160	71.1±6.7/70.7±6.2	12∶26/12∶24	YA-60BB/VA-60BB
Wirtitsch 2009 [20]	Austria	24	48	74±8	6∶18	YA-60BB/VA-60BB
Barisić 2007 [21]	Croatia	60	120	68±4.5/67±4.2	9∶21/9∶21	Acrysof Natural IOL/AcrySof MA60BM IOL
Marshall 2005 [17]	England	297	594	≥60 (0.3/1.0)	29.3∶70.7/39.5∶60.5	SN60AT/SA60AT
Bhattacharjee 2006 [22]	India	13	26	62.15±6.68	6∶7/6∶7	SN60AT/SA60AT
Schmidinger 2008 [23]	Austria	31	62	73.40±7.64	NA	YA-60BB/VA-60BB
Mester 2008 [24]	Germany	47	94	Range 50–80	NA	YA-60BB/VA-60BB
Leibovitch 2006 [25]	Australia	19	19	74±6/74±6	3∶6/6∶4	SN60AT/SA60AT
Vuori 2006 [26]	Finland	37	52	72±8/73±7	NA	SN60AT/SA60AT
Rocha 2007 [27]	Brazil	40	80	71.0/69.2	2∶3	SN60AT/Sensar AR40
Caporossi 2009 [28]	Italy	50	100	70.2±4.1/68.4±5.1	NA	SN60AT/Sensar AR40
Caporossi 2007 [29]	Italy	50	100	70.2±4.1/68.4±5.1	NA	SN60AT/Sensar AR40
Pandita 2007 [30]	India	80	80	61±2.7/59±3	51∶49/52∶48	SN60AT/SA60AT
Neumaier-Ammerer 2010 [31]	Austria	80	80	NA	NA	YA-60BB/VA-60BB & SN60AT/SA60AT
Wang 2010 [32]	China	79	79	67±8	NA	YA-60BB/MC611

NA, not available.

**Table 2:** Characteristics of RCTS (n = 15) included in the meta-analysis.

First author (date)	Measurement			Follow-up	Loss
	Visual acuity	Contrast sensitivity	Color Vision		
Hayashi 2006 [14]	*	* CAT-2000	NA	3 m	6 of 80 (7.5%)
Wirtitsch 2009 [20]	*	* Holladay	* Lanthony D-15	90±10 d	No
Barisić 2007 [21]	*	NA	NA	6 m	No
Marshall 2005 [17]	*	* CSV-1000E	* FM??? D-15	1 y	86 of 297 (28.96%)
Bhattacharjee 2006 [22]	*	* Pelli-Robson	* FM 100-hue	18 m	No
Schmidinger 2008 [23]	*	* Moorfields	NA	12 w	3 of 31 (9.68%)
Mester 2008 [24]	*	* ETDRS???+FACT???	* FM 100-hue	12 m	8 of 47 (17.02%)
Leibovitch 2006 [25]	*	* Pelli-Robson	* FM D-15	6 m	No
Vuori 2006 [26]	*	NA	* FM 100-hue	6 m	No
Rocha 2007 [27]	*	NA	NA	90 d	10 of 80 (12.5%)
Caporossi 2009 [28]	*	* Optec 6500	NA	2 y	6 of 50 (12%)
Caporossi 2007 [29]	*	* Optec 6500	NA	2 m	No
Pandita 2007 [30]	*	* CSV-1000E	NA	3 m	7 of 80 (8.75%)
Neumaier-Ammerer 2010 [31]	*	* CSV-1000E + Pelli-Robson	* Roth 28-hue	2 m	4 of 80 (5%)
Wang 2010 [32]	*	* ETDRS + Optec 6500	* FM 100-hue	3 m	No

*, yes; d = days; m = months; y = years; NA, not available.

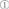
 ETDRS, Early Treatment Diabetic Retinopathy Study contrast charts;

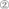
 FACT, Functional acuity contrast test;

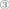
 FM, Farnsworth-Munsell.

### 3. Quality assessment

**3.1. Sequence generation:** In 6 of all the RCTs included in the systematic review, the investigators described a random component in the sequence generation process such as: referring to a random number table [14] or using a computer random number generator [30] or shuffling envelopes [25], [26], [31]. The remainder did not describe the specific methods of random sequence generation.

**3.2. Masking:** Of 13 studies that described their masking or binding, 1 used triple-blinding [30], 8 used double-blinding [14], [20], [22]–[24], [26], [31], [32] and 4 used single-blinding [17], [21], [25], [29].

**3.3. Withdrawals:** All studies described the data of missing patients. Among these, 8 studies had missing cases: 6 of 80 (7.5%) [14]; 86 of 297 (28.96%) [17]; 3 of 31 (9.68%) [23]; 8 of 47 (17.02%) [24]; 10 of 80 (12.5%) [27]; 6 of 50 (12%) [28]; 7 of 80 (8.75%) [30]; and 4 of 80(5%) [31].

**3.4. Other sources of bias:** Only 2 studies used the following or an equivalent method to achieve allocation concealment: sealed envelopes [26] or sequential numbering [14].

Eight studies [14], [17], [20]–[22], [24], [25], [32] described the patients' subjective satisfaction of visual quality between the blue light-filtering IOL and UV light-filtering IOL after surgery, and the other studies did not. Therefore, we do not know the existence of other potential factors.

The quality assessment of included studies is shown in Table 3.

**Table 3:** Evaluation of the quality of RCTs included in the meta-analysis.

Study	Sequence generation	Double-blind	Withdrawals	Jadad score(0–5)
Hayashi 2006 [14]	Adequate	Adequate	DS	5
Wirtitsch 2009 [20]	Adequate	Adequate	DS	5
Barisić 2007 [21]	UA	SB	DS	2
Marshall 2005 [17]	UA	SB	DS	2
Bhattacharjee 2006 [22]	UA	Adequate	DS	4
Schmidinger 2008 [23]	UA	Adequate	DS	4
Mester 2008 [24]	UA	Adequate	DS	4
Leibovitch 2006 [25]	Adequate	SB	DS	3
Vuori 2006 [26]	Adequate	Adequate	DS	5
Rocha 2007 [27]	UA	NA	DS	2
Caporossi 2009 [28]	UA	NA	DS	2
Caporossi 2007 [29]	UA	SB	DS	2
Pandita 2007 [30]	Adequate	Adequate	DS	5
Neumaier-Ammerer 2010 [31]	Adequate	NA	DS	4
Wang 2010 [32]	UA	Adequate	DS	4

UA, Unclear; SB, Single blinding; DB, Double blinding; TB, Triple blinding; DS, Described; NA, not available.

### 4. Efficacy analysis

**Figure 2:**
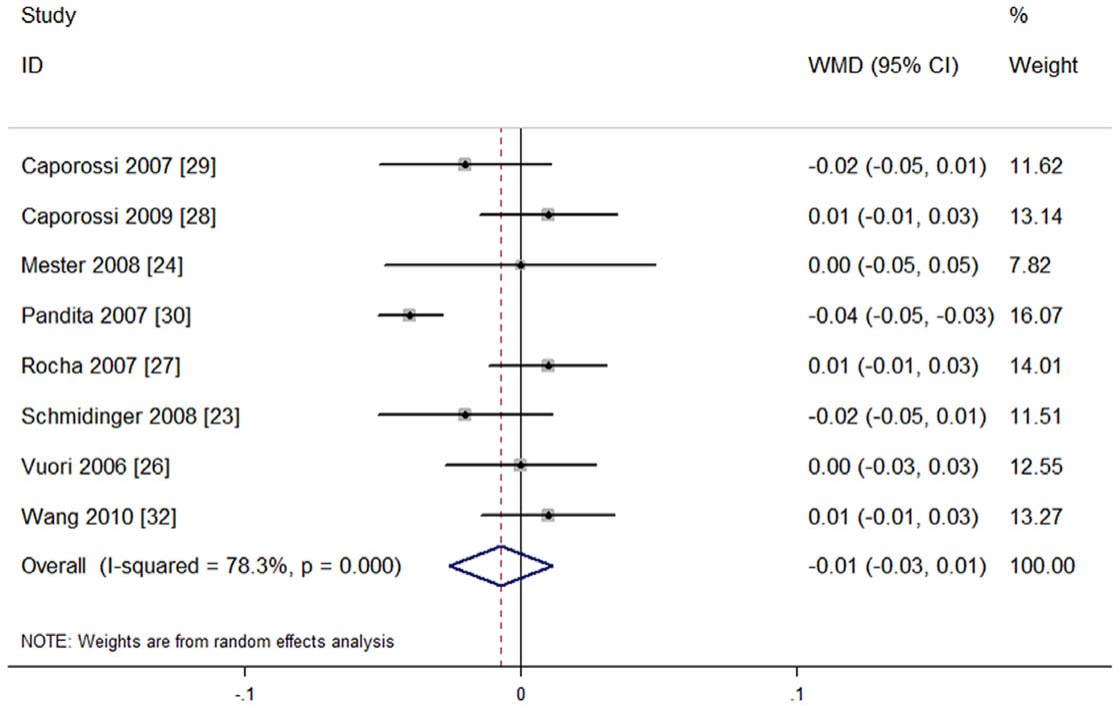
Meta-analysis of postoperative best corrected visual acuity (BCVA).

**4.1. Postoperative best corrected visual acuity (BCVA):** All studies described postoperative BCVA; the results of 8 studies [23], [24], [26]–[30], [32] (596 eyes) were LogMAR transformation, including mean (m), standard deviation (SD) and sample size (n). Also, these studies had heterogeneity of effect size (P = 0.0001, I^2^ = 78.3%), so the random effect model was used for meta-analysis. The results are shown in Figure 2. No significant difference between the two groups [WMD = −0.01, 95%CI (−0.03, 0.01), P = 0.46], indicated that blue light-filtering IOL groups and UV light-filtering IOL groups were not significantly different in postoperative BCVA.

**Figure 3:**
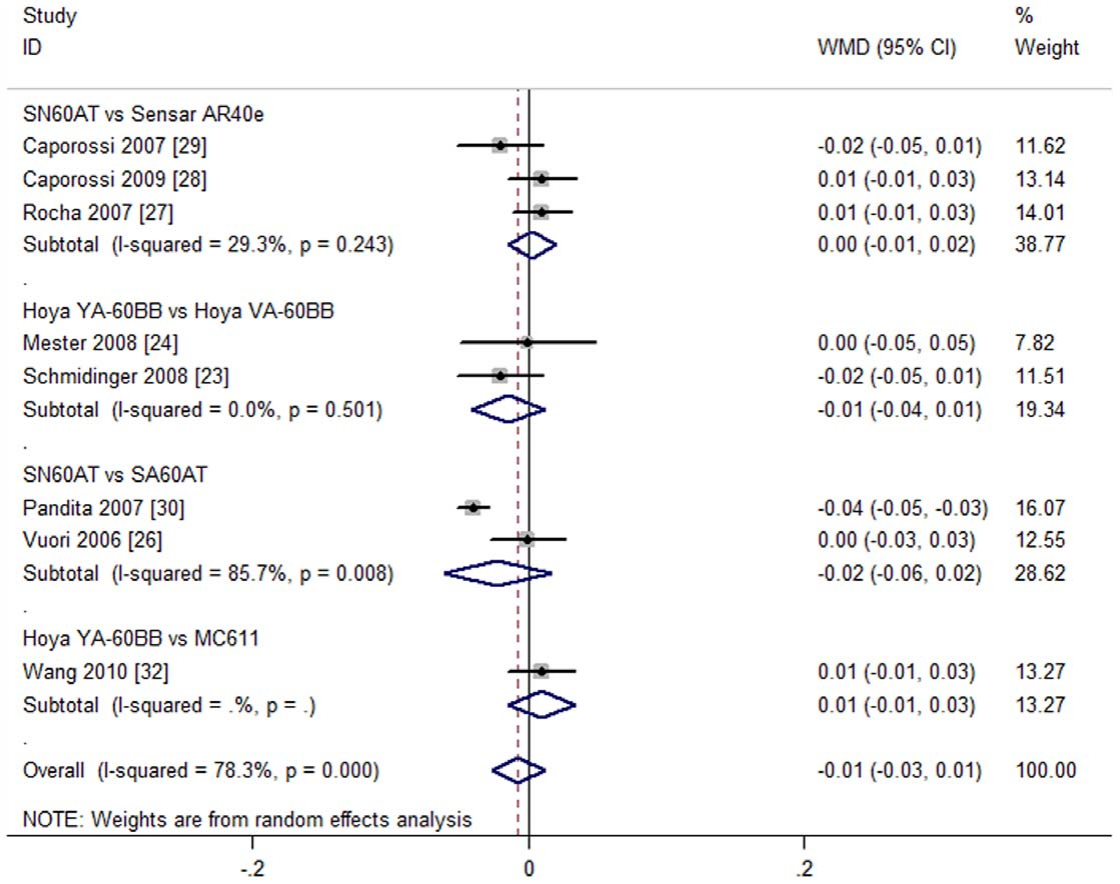
Subgroup analysis of postoperative best corrected distance visual acuity (BCVA).

Subgroup analysis was conducted at the same time, according to the different IOL types. The studies were divided into four subgroups: blue light-filtering AcrySof Natural SN60AT IOL vs. UV light-filtering Sensar AR40e IOL (Subgroup 1) [27]–[29]; blue light-filtering Hoya YA60BB vs. UV light-filtering VA60BB (Subgroup 2) [23], [24]; blue light-filtering AcrySof Natural SN60AT IOL vs. UV light-filtering AcrySof SA60AT IOL (Subgroup 3) [26], [30]; and blue light-filtering Hoya YA60BB vs. UV light-filtering MC61131 (Subgroup 4) [32]. The results are shown in Figure 3.

All four subgroups were used to produce the random effect model for BCVA, and the results obtained from any subgroup show no significant differences. The results are as follows: Subgroup 1 (SN60AT vs. AR40e IOL, three studies [27]–[29], recruiting 258 eyes) [WMD = 0.00, 95%CI (−0.01, 0.02), P = 0.763]; Subgroup 2 (YA60BB vs. VA60BB, two studies [23], [24], recruiting 134 eyes) [WMD = −0.01, 95%CI (−0.04, 0.01), P = 0.294]; Subgroup 3 (SN60AT vs. SA60AT, two studies [26], [30], recruiting 125 eyes) [WMD = −0.02, 95%CI (−0.06, 0.02), P = 0.27]; Subgroup 4 (YA60BB vs. MC611, one study [32], recruiting 79 eyes) [WMD = 0.01, 95%CI (−0.01, 0.03), P = 0.42]. All the results indicated that blue light-filtering IOL groups and UV light-filtering IOL groups were not significantly different in postoperative BCVA.

**4.2. Postoperative contrast sensitivity:** Twelve studies [14], [17], [20], [22]–[25], [28]–[32] compared the contrast sensitivity of blue light-filtering IOLs and UV light-filtering IOLs after implantation. The methods of assessing contrast sensitivity were described in Table 2. However, most of the literature using several different methods reported only the contrast sensitivity graph or P value, but no detailed data. On the other hand, for part of the data in reports, means and standard deviations cannot be calculated, and there was failure to pool analysis. Although we tried to contact the author, results of the corresponding data were not obtained. Thus, these could cause measurement bias. Thereafter, we conducted a descriptive analysis with results shown in Table 4.

**Table 4:** Comparison of postoperative contrast sensitivity in two groups.

		Photopic Conditions		Mesopic Conditions	
Model of IOLs	Study	With Glare	Without Glare	With Glare	Without Glare
SN60AT/SA60AT	Marshall 2005 [17]	NA	*	NA	*
	Pandita 2007 [30]	NA	*	*	*
	Neumaier-Ammerer 2010 [31]	*	*	*	*
SN60AT/Sensar AR40	Caporossi 2009 [28]	NA	*	NA	*
	Caporossi 2009 [29]	NA	*	NA	*
	Hayashi 2006 [14]	*	*	*	*
	Wirtitsch 2009 [20]	The blue light-filtering IOLs had worse contrast sensitibity compared with the UV-filtering IOL.			
YA-60BB/VA-60BB	Schmidinger 2008 [23]	NA	*	NA	NA
	Mester 2008 [24]	NA	*	*	*
	Neumaier-Ammerer 2010 [31]	*	*	*	*
YA-60BB/MC611	Wang 2010 [32]	*	*	*	*

* , No statistically significant difference; NA, not available.

**Figure 4:**
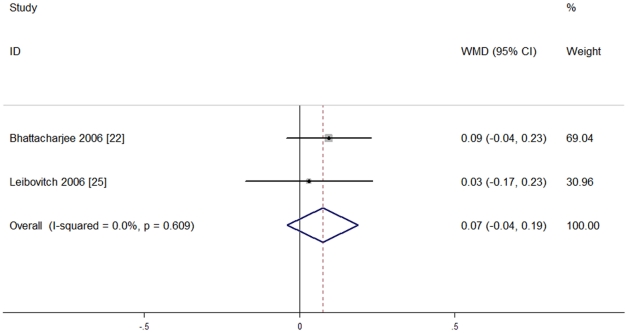
Meta-analysis of postoperative contrast sensitivity.

Only two studies [22], [25] recruiting 45 eyes used the same measurement method (Pelli-Robson contrast sensitivity chart), and reported complete data. They had no heterogeneity of effect size (P = 0.609, I^2^ = 0%), so the fixed effect model was used for meta-analysis. No significant difference between the two groups was seen [WMD = 0.07, 95%CI (−0.04, 0.19), P = 0.20], indicating that blue light-filtering IOL groups and UV light-filtering IOL groups were not significantly different in postoperative contrast sensitivity. The results are shown in Figure 4.

**4.3. Postoperative color vision:** Eight studies [17], [20], [22], [24]–[26], [31], [32] compared the color vision of blue light-filtering IOLs and UV light-filtering IOLs after implantation. The methods of assessing color vision were described in Table 2. Five of them [24]–[26], [31], [32] reported complete data, and one [24] reported that color vision for blue with the blue light-filtering IOL was significantly reduced under photopic and mesopic conditions; another study [32] reported that the UV light-filtering IOL had significantly better color vision than the blue light-filtering IOL under mesopic conditions. The remaining studies showed no statistically significant difference.

**Figure 5:**
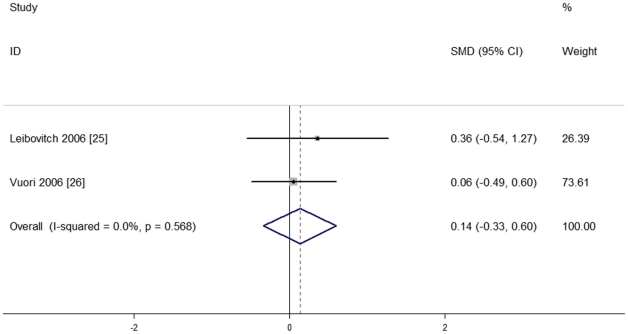
Meta-analysis of postoperative overall color vision.

Two studies [25], [26] recruiting 71 eyes used different scales to report their outcomes in overall color vision, so that standardized mean differences (SMD) were more appropriate. They also had no heterogeneity of effect size (P = 0.59, I^2^ = 0%), so the fixed effect model was used for meta-analysis. No significant difference between the two groups was seen [SMD = 0.14, 95%CI (−0.33, 0.60), P = 0.566], indicating that blue light-filtering IOL groups and UV light-filtering IOL groups were not significantly different in postoperative color vision. The results are shown in Figure 5.

**Figure 6:**
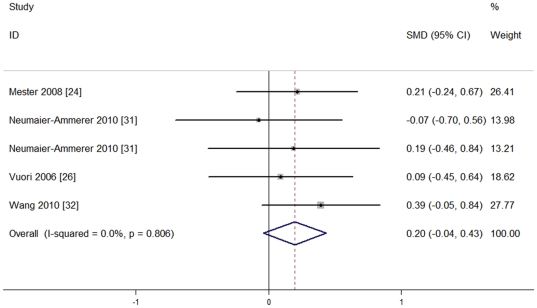
Meta-analysis of postoperative color vision in the blue light spectrum under photopic light condition.

Four studies [24], [26], [31], [32] recruiting 281 eyes tested postoperative color vision in the blue light spectrum under photopic light conditions. They used different scales to report their outcomes, so standardized mean differences (SMD) were more appropriate. Also, they had no heterogeneity of effect size (P = 0.806, I^2^ = 0%), so the fixed effect model was used for meta-analysis. No significant differences were seen between the two groups [SMD = 0.20, 95%CI (−0.04, 0.43), P = 0.099], indicating that blue light-filtering IOL groups and UV light-filtering IOL groups were not significantly different in postoperative color vision in the blue light spectrum under photopic light conditions. The results are shown in Figure 6.

**Figure 7:**
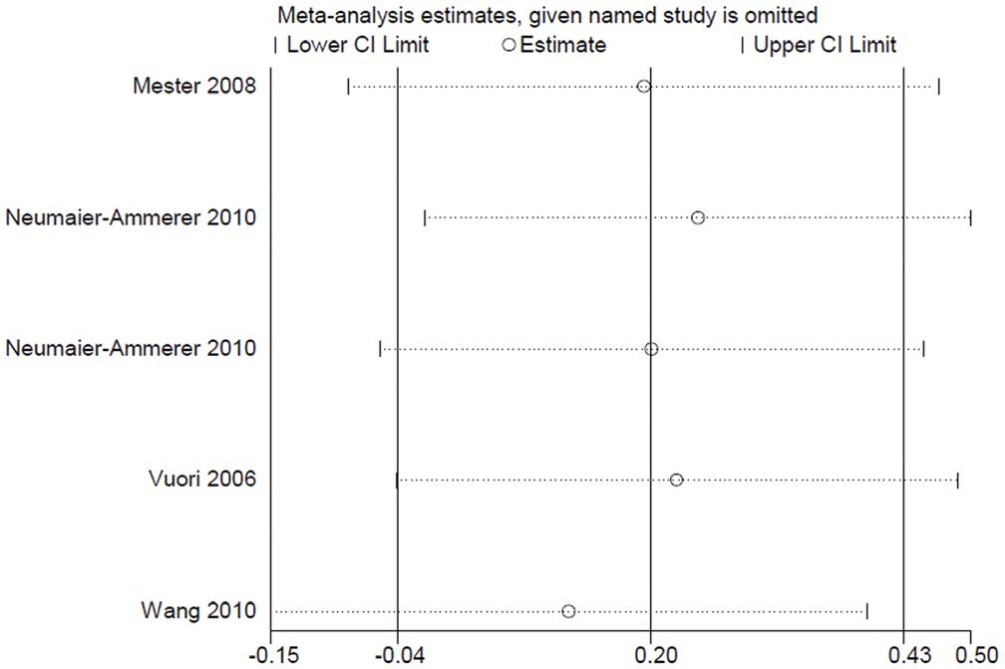
Sensitivity analysis of postoperative color vision in the blue light spectrum under photopic light condition.

In addition, a sensitivity analysis suggested that the result was stable after deleting anyone's particular outcome. The results are shown in Figure 7.

**Figure 8:**
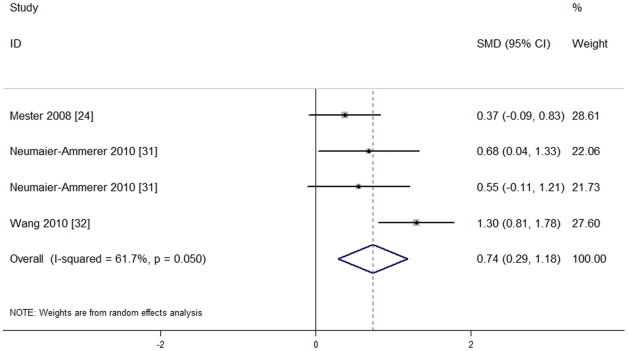
Meta-analysis of postoperative color vision in the blue light spectrum under mesopic light condition.

Three studies [24], [31], [32] recruiting 229 eyes tested postoperative color vision in the blue light spectrum under mesopic light conditions. They used different scales to report their outcomes, so standardized mean differences (SMD) were more appropriate. Also, they had no heterogeneity of effect size (P = 0.05, I^2^ = 61.7%), so the random effect model was used for meta-analysis. There was a significant difference between the two groups [SMD = 0.74, 95%CI (0.29, 1.18), P = 0.001], indicating that color vision for blue with blue light-filtering IOLs was significantly reduced under mesopic conditions. The results are shown in Figure 8.

**Figure 9:**
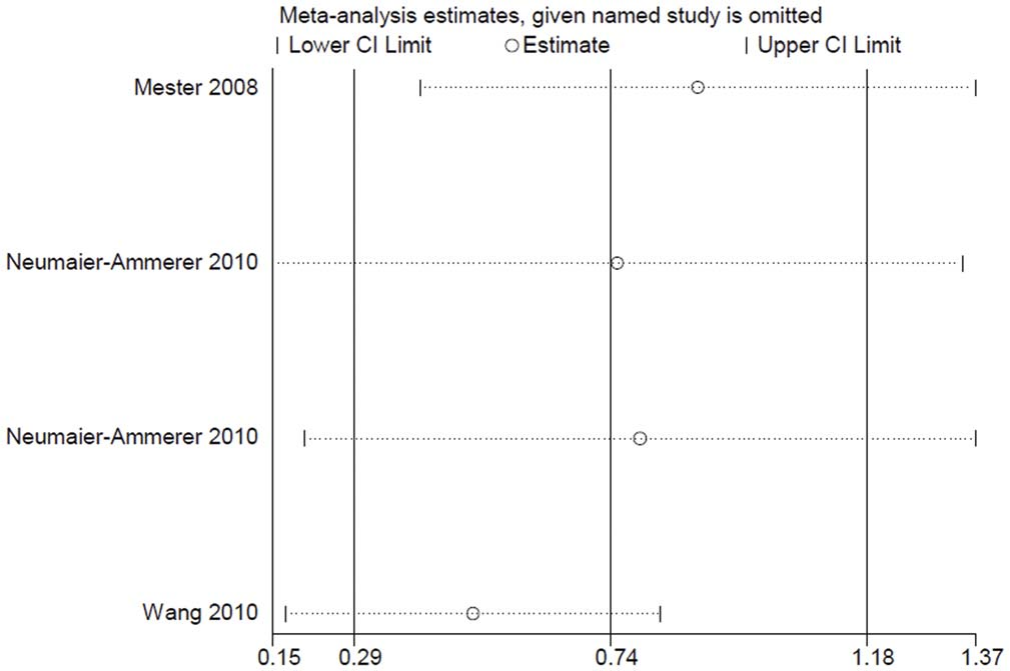
Sensitivity analysis of postoperative color vision in the blue light spectrum under mesopic light condition.

Additionally, the sensitivity analysis revealed that color vision with blue light-filtering IOLs was significantly reduced under mesopic conditions in the blue light spectrum. The results are shown in Figure 9.

**4.4. Postoperative subjective satisfaction of visual quality and adverse visual events:** Four studies [14], [20], [21], [23] reported that different patients' subjective satisfaction of visual quality between the blue light-filtering IOL and UV light-filtering IOL after surgery. In the two studies [14], [20] that used questionnaires for evaluation, Wirtitsch and associates [20] using a questionnaire regarding color vision or contrast vision or vision under poor light conditions, they reported that 3 of 24 patients noticed a difference and all could correctly identify the eye implanted with the blue light-filtering IOL. However, all 3 patients said that they were not disturbed in binocular vision. In the report by Hayashi and associates [14], where patients were given a standardized questionnaire regarding glare symptoms and cyanopsia, the authors found that the incidence of patients who noticed cyanopsia was significantly less in the blue light-filtering IOL group than in the UV light-filtering IOL group at 2 weeks after surgery (p = 0.0234), but no patients reported cyanopsia at 3 months. In addition, another two studies [21], [23] reported different patients' subjective satisfaction and subjective color vision in the left eye and right eye. Barisić and associates [21] described high satisfaction in patients who were implanted with a blue light-filtering IOL. Schmidinger and associates [23] found that 2 patients independently reported subjective changes in color vision in the eye with the blue light-filtering IOL; the changes probably resulted from the difference in color brightness because yellow IOLs reduce color brightness in the blue range of visible light. Furthermore, four studies [17], [24], [25], [32] found no significant differences in subjective satisfaction of visual quality or lens-related adverse events in either group after surgery, which only Wang and associates [32] used the standardized questionnaire regarding subjective evaluation of glare, halo, and color vision perception. The remaining studies reported no relevant results in this regard.

## Discussion

This systematic review compared the postoperative visual performance of RCTs between blue light-filtering IOLs and UV light-filtering IOLs, with the results showing that no statistically significant differences were found in comparing postoperative best corrected visual acuity, contrast sensitivity, and overall color vision. However, blue light-filtering IOLs demonstrated some compromise in the blue light spectrum under mesopic light conditions.

Large-scale epidemiological and animal studies have demonstrated that exposure to short wavelength visible light may be associated with a potential risk for accelerating the pathology of age-related macular degeneration (AMD) and retinal damage [2], [3], [33]–[35]. The natural aging crystalline lens can absorb amounts of UV and visible light, due to natural yellowing and opacification, going against the phototoxicity-AMD hypothesis [5]. Evidence suggests that blue light can damage the macula in patients who have undergone cataract surgery or clear lens extraction [6]. Therefore, an ideal IOL should be similar to that of the adult crystalline lens. Blue light-filtering IOLs show transmittance curves similar to that of a 53-year-old person's natural crystalline lens to help reduce the potential damage from blue light reaching the retina [7].

The implantation of blue light-filtering IOLs caused debate over their potential negative effects on postoperative visual performance, such as BCVA, contrast sensitivity and color vision, especially in dark conditions. We found that most of the literature overwhelmingly demonstrated that there were no detrimental effects of blue light-filtering IOLs on clinical visual recovery, which was consistent with our results. However, Mester and associates [24], in one of the included studies, mentioned that color vision in the blue light spectrum with the blue light-filtering IOLs (HOYA YA60BB) was significantly reduced under mesopic and photopic conditions: the impairment did not exceed the normal range or induce subjective disturbance of color vision. Furthermore, Barisić and associates [21] indicated that the blue light-filtering IOLs (HOYA YA60BB) had inferior contrast acuity and foveal threshold compared with the UV light-filtering IOLs. Unfortunately, we could not obtain complete data for a pooled analysis. Wang and associates [32] reported that blue light-filtering IOLs gave poor contrast sensitivity and color vision under mesopic conditions, which was in agreement with the conclusions of Neumaier-Ammerer and associates [31]. In fact, blue light-filtering IOLs have been shown to reduce the incidence of photophobia and cyanopsia in the early postoperative period [11], [36].

Meta-analysis is the pooling of data from a number of different studies and objectively reanalyzing the resulting data set to provide a more precise result to assist in making clinical decisions. A possible limitation includes inappropriate pooling of data and publication bias. In our analysis, to avoid acknowledged and unintended duplication of data, we included only the most recent series of patient groups and randomized controlled trials, which are the optimal choice for meta-analyses. To minimize the publication bias, we conducted an electronic search and a manual search of the references of relevant studies to identify all the potential articles. This systematic review included 15 studies, all of which met the strict inclusion criteria; therefore, the intervention groups and control groups were comparable. However, the overall quality of the studies was not high. Only six studies had adequate sequence generation, two studies used adequate methods to achieve allocation concealment, and 2 studies made no mention of masking. In addition, different follow-up times and less reporting of postoperative visual adverse events could cause selection bias. Several studies lacked sufficient data for analysis, or involved different measurement methods, or used different units of measurement, or not used a standard questionnaire to assess: all of these could cause measurement bias. All the information in this systematic review came from the published literature, with special reports or unpublished data not being included, as they could cause publication bias. Therefore, to improve the quality of RCTs in the future, further verification of the postoperative visual performance and safety of blue light-filtering IOLs is needed. The format of reports should comply with the rules of CONSORT, providing detailed and transparent information in order to judge the authenticity of the outcomes.

In conclusion, our systematic review, as well as other clinical reports, suggests that the blue light-filtering IOLs had postoperative visual performance comparable to the UV light-filtering IOLs, but conclusions regarding color vision are still inconsistent.
